# The frequency of missed test results and associated treatment delays in a highly computerized health system

**DOI:** 10.1186/1471-2296-8-32

**Published:** 2007-05-22

**Authors:** Terry L Wahls, Peter M Cram

**Affiliations:** 1 Iowa City Department of Veterans Affairs (VA) Medical Center, Iowa City, IA, USA; 2 Division of General Medicine, Department of Internal Medicine, University of Iowa Carver College of Medicine, Iowa City, IA, USA; 3 Center for Research in the Implementation of Innovative Strategies in Practice (CRIISP) VA HSR&D Center of Excellence, Iowa City Department of Veterans Affairs (VA) Medical Center, Iowa City, IA, USA

## Abstract

**Background:**

Diagnostic errors associated with the failure to follow up on abnormal diagnostic studies ("missed results") are a potential cause of treatment delay and a threat to patient safety. Few data exist concerning the frequency of missed results and associated treatment delays within the Veterans Health Administration (VA).

**Objective:**

The primary objective of the current study was to assess the frequency of missed results and resulting treatment delays encountered by primary care providers in VA clinics.

**Methods:**

An anonymous on-line survey of primary care providers was conducted as part of the health systems ongoing quality improvement programs. We collected information from providers concerning their clinical effort (e.g., number of clinic sessions, number of patient visits per session), number of patients with missed abnormal test results, and the number and types of treatment delays providers encountered during the two week period prior to administration of our survey.

**Results:**

The survey was completed by 106 out of 198 providers (54 percent response rate). Respondents saw and average of 86 patients per 2 week period. Providers encountered 64 patients with missed results during the two week period leading up to the study and 52 patients with treatment delays. The most common missed results included imaging studies (29 percent), clinical laboratory (22 percent), anatomic pathology (9 percent), and other (40 percent). The most common diagnostic delays were cancer (34 percent), endocrine problems (26 percent), cardiac problems (16 percent), and others (24 percent).

**Conclusion:**

Missed results leading to clinically important treatment delays are an important and likely underappreciated source of diagnostic error.

## Background

There is growing evidence that delays in diagnosis constitute a common medical error and represent a significant threat to patient safety [1-5]. Yet the precise causes of these diagnostic delays and a comprehensive portrait of the magnitude of the problem remain elusive. Available data suggest that problems in the test result reporting system, often related to the mishandling of abnormal test results ("missed results"), contribute to the majority of diagnostic delays [6,7]. Little however is known about the epidemiology and clinical consequences of mishandled abnormal test results [8].

A number of isolated studies have examined the incidence of missed results within discrete healthcare systems by focusing on individual tests (e.g., DXA scans, mammograms) [9-13]. Indeed these studies have demonstrated that clinically important abnormal test results are lost to follow up more frequently than is generally appreciated. However, by narrowly focusing upon individual tests in great detail, these studies were unable to estimate the overall burden of all missed results across a broad population of patients. Studies quantifying the incidence of missed results among populations of patients are far more limited.

An important study in this regard was conducted by Roy et al, who examined clinician awareness of significantly abnormal test results which had returned after the patient's discharge from a large academic center. The investigators found that clinically important missed results occurred in 0.9 percent of patient discharges [14]. In another study, this one of primary care ambulatory practices, clinicians documented test results were missing, often causing clinical delay, for one out of ten patients in clinic [15]. Also serial surveys of primary care providers, documented nearly have of the clinicians had encountered patients having clinically significant missed results in prior two weeks of clinic [16]. While these studies provide evidence that missed test results are common, the epidemiology of missed results within a population remains unknown.

The Veterans Health Administration (VHA), with over 5 million patients, 22 regional health care networks and hundreds of integrated healthcare delivery systems all linked by a common electronic medical record (EMR) has long been recognized as a leader in quality and patient safety. Equipped with an advanced EMR which integrates laboratory, radiology and clinical notes and provides the capability of making test result information available within the EMR as soon as it has been finalized by the diagnostic service, the VHA presents an opportunity to examine the epidemiology of missed results in a healthcare system that has already implemented many technologies designed to minimize this problem.

In an effort to determine both the incidence and clinical significance of missed test results in the VHA, we built upon our previous work to study the frequency and types of missed results and associated treatment delays that providers encountered in their clinical practices.

## Methods

### Participants and setting

We administered a survey (described below) to primary care providers (126 staff physicians and 72 mid-levels i.e. physician assistants, and nurse practitioners) practicing within VA Midwest Health Care Network encompassing Minnesota, Iowa, Nebraska, South Dakota, and North Dakota. This health care system, also known as Veterans Integrated Service Network 23 (VISN 23), includes three large academic medical centers, five smaller community and rural hospitals, and numerous smaller community-based outpatient clinics.

### Survey development

This survey was developed as part of an ongoing quality improvement initiative assessing test result reporting practices and associated problems in VISN 23. A multi-disciplinary task force, consisting of primary care clinicians, specialty clinicians, radiology and pathology clinicians and administrators developed the first results reporting survey, to explore problems in the test result reporting system which was initially fielded in May 2005 and first identified that nearly half of the providers had encountered missed results and over a third had encountered treatment delays [16]. We took the survey questions from this survey, added additional questions to gather information about the types of missed results and associated treatment delays, processes used to ensure follow up, and provider support for potential interventions to decrease the frequency of missed results to create the survey which was used for this study. The questions were reviewed for readability and clarity by members of the task force and affiliate general medicine faculty.

We obtained limited demographic information and clinical effort to provide estimates of the patient volume. Although we asked in which healthcare system the clinician practiced, we did not obtain other demographic data in order to protect respondent privacy. Next we collected information about the number of days each respondent spent in clinic in the prior two weeks and the typical number of patients seen per session. See Appendix for the list of questions.

The medical error section of the survey asked about missed test results and resultant treatment delays encountered by respondents during the two week period prior to receipt of the survey. Providers were asked to specify "how many patients they had encountered during the prior two weeks with an abnormal result that had been missed because it had not received the anticipated clinical response from the ordering service (limit to greater than one month delay or such a critical result than a month delay would have been inappropriate)." Subsequently they were then asked to choose from a list, which study or studies were missed (e.g., imaging, anatomic pathology, fecal occult blood test (FOBT), prostate specific antigen (PSA), etc.). A follow up question asked the respondent to specify how many patients they had encountered in the prior two week period whom "may have experienced a delay in either diagnosis or treatment due to a missed diagnostic result that was overlooked by the ordering service." Again, in follow up, they were then asked to choose from a list, which type of treatments or diagnoses were delayed.

In the next section, because primary care clinicians had expressed concern that patients frequently scheduled visits with them expressly to obtain results of tests that had been ordered by specialists in our initial survey, we asked respondents how many patients they had seen in the prior two weeks because a patient asking a specialty clinic about their test results had been redirected to primary care (a.k.a., a patient diversion). Two supplemental questions, using a 5 point Likert scale (ranging from 1 [strongly agree] to 5 [strongly disagree]) investigated the time such visits took and whether the provider felt competent to interpret test results in those circumstances.

The fourth section asked about procedures and processes providers used to avoid missing test results in their practice. To provide the reader an understanding of VA EMR, the notifications processes within the EMR as they existed in the network at the time of the survey are summarized. In an effort to decrease the volume of notifications that providers see each day, providers were given control of the settings determining which clinical laboratory result and which radiology result notifications were presented at provider sign on, i.e. all results, only abnormal results, or only critical results as defined by the hospital clinical executive board. Since paper copies of test results were largely eliminated, the notifications within the EMR were generally the only means by which a provider received copies of test results.

In particular, providers were asked whether they used any of the following procedures: 1) Notifications within the electronic medical record (EMR) set to receive all test results; 2) Notifications set to receive only abnormal results; 3) Notifications which flag only the most critical test; 4) Paper based log of tests ordered; 5) Delegation of responsibility to support staff; and 6) Other systems.

Because we also wanted to know how providers ensured patients had completed follow up after an abnormal result, the fifth section asked the providers to select from a list of options, which was the best description of their usual practice. Two active processes i.e. not dependent upon the patient action, were presented: use of an electronic or paper log or staff monitor. The three passive processes, i.e. dependent on the patient action, were presented: instructing patient to call if follow up did not occur, review of previous clinic note when patient returns, and no processes in place.

Finally, we asked providers to rate the "helpfulness" of eight potential interventions in the VA results management system designed to improve test result management (using a 4 point Likert scale ranging from 1{probably very helpful} to 4{definitely more disruptive than helpful}). Potential changes included:

1. Establishing the expectation for patients that all test results will be reported to them.

2. Providing copies of all diagnostic test results directly to patients.

3. Providing, to the ordering service, summary monthly reports of abnormal labs specific to a diagnosis group (e.g. patients with CAD and LDL>110 or CXR with possible mass).

4. Periodic summary reports of patients with abnormal test results that have not received the anticipated clinical response (e.g. abnormal mammograms or elevated PSA).

5. Establishment of a consistent process or procedure (SOP) for the "hand off" of diagnostic test results when a provider is absent or leaves the service.

6. The establishment of a consistent SOP for results management and reporting by each clinical service.

7. A convenient process for providers to generate results letters to patients.

8. A secure voice messaging system to patients for results reporting and instructions from providers.

### Survey administration

The providers were sent an email two weeks prior to the survey, briefly noting the problem of missed results and associated treatment delays, reviewing the network's commitment to periodic assessment of missed results related issues which would be conducted again during the next month. Two weeks later, the providers with more than 450 continuity patients were sent invitations to participate in the survey. Over the next three weeks, three reminder e-mails were sent, thanking providers who had completed the survey and encouraging those who had not yet completed the survey to do so.

### Data analysis

The data from the above survey was collected via a secure internet web site. Survey responses were used to calculate the mean number of patients seen per provider per week and the proportion of patients who experienced missed test results and delays in diagnosis or treatment using Microsoft Excel (Microsoft, Inc. Redmond, WA) and Stata SE Version 8.2 (Stata Corp., College Station, TX). These analyses were approved by the University of Iowa Institutional Review Board.

## Results

The survey was completed by 106 of 198 providers for an overall response rate of 54 percent. The response rate from the eight participating health care system ranged from a low of 40 percent (8 of 20) to a high of 69 percent (11 of 16). Providers reported working in clinic an average of 8.3 of a possible 10 days during the two week period prior to the survey administration. Approximately 9100 patient encounters were reported by the respondents with each provider on average seeing 86 patients in the prior two weeks.

**Figure 1:**
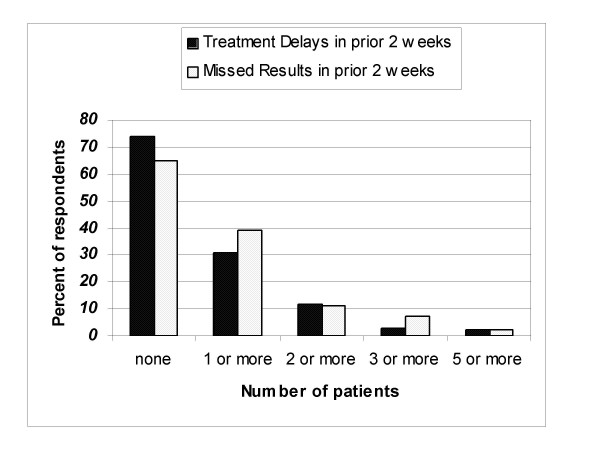
The number of missed results or treatment delays associated with missed results encountered by providers.

**Figure 2:**
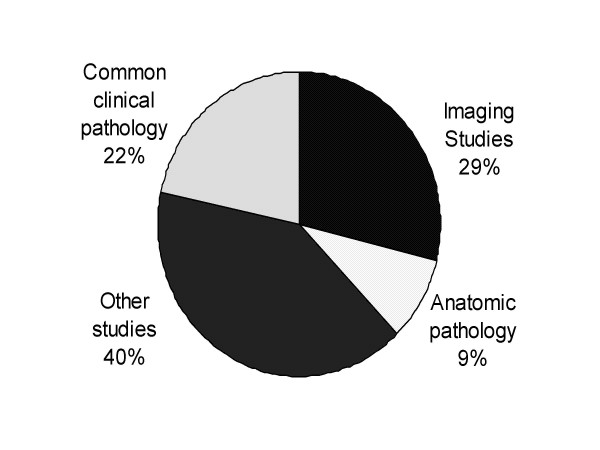
Distribution of the diagnostic studies missed.

During this period, 63 percent of survey respondents reported that they did not encounter any patients with a probable missed result while 37 percent reported encountering at least one patient with a missed result (Figure 1). The types and distribution of the diagnostic studies providers reported encountering as a missed result are summarized in Figure 2. Clinical laboratory tests (specifically fecal occult blood test (FOBT), prostate specific antigen (PSA), glucose, hemoglobinA1c, and lipids) and imaging studies were the most commonly reported missed results, and together accounted for 51 percent of all missed results. The diagnostic studies most likely to be related to a potential malignancy (FOBT, PSA, anatomic pathology, mammograms, and chest X-ray) accounted for 35 percent of the missed results. The category other included laboratory studies such as microbiology, send out clinical laboratory studies, diagnostic procedures such as echocardiograms, pulmonary function studies, and other non-specified studies.

**Figure 3:**
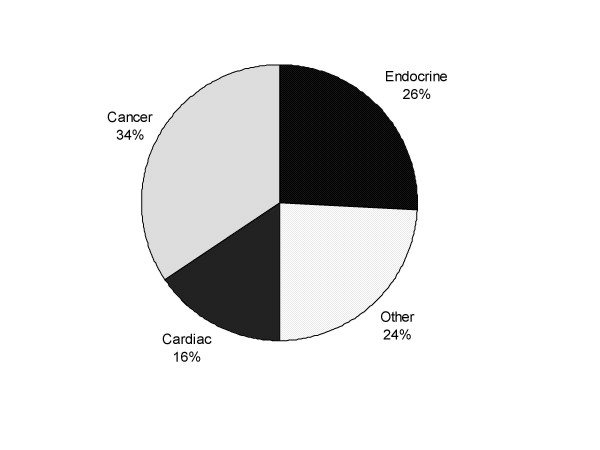
Distribution of treatment delays associated with missed results.

In the follow up question about treatment delays, a total of 32 providers (30 percent of respondents) reported encountering one or more patients (a total of 52 patients in all) with delays in diagnosis or treatment due to missed test results. The types of diagnoses and treatments that were reported are shown in Figure 3. Cancer, of which 2/3 were prostate, colorectal or lung cancer, was the most frequently reported treatment delay (34 percent) followed by endocrine disorders (i.e. diabetes, hypothyroidism, hyperlipidemia at 26 percent) cardiac disorders (acute cardiac diagnosis or adjustment of therapy at 16 percent), and other diagnosis or treatments (30 percent).

One or more patient diversions in the prior clinic session were reported by 42 percent of the providers, accounting for 7 percent of the primary care visits. The majority (70 percent) either strongly agreed or agreed that "the time lost as a result of investigating the test is very burdensome to my practice" and just under half (46 percent) either strongly agreed or agreed that "they generally did not know the clinical significance of the diagnostic tests they were asked to provide."

### Procedures used to manage results and follow up for abnormal results

Providers reported using a wide array of processes to avoid missing test results. The majority of providers (55 percent) reported reliance on the electronic notification system (i.e. electronic "in box") within the EMR with settings customized either to receive all results (31 percent), abnormal results only (21 percent) or reliance on specific "order flags" for the most important tests placed (3 percent). Other process providers reported using included a combination of both paper based logs and notifications within the EMR (34 percent), delegation to support staff (3 percent), and paper based log (8 percent).

### Ratings of helpfulness of interventions

The three interventions that respondents rated most favorably to enhance the management of test results were: 1) establishment of a standard procedure to manage results during the absence of the ordering provider (mean Likert score 1.57; SD 0.310); 2) electronic verification of provider review of results (mean Likert score 1.63; SD 0.21); and, 3) establishment of standard procedures for managing results for the clinical service (mean Likert score 1.68; mean SD 0.24). See Table 1.

**Table 1:** Summary of Provider Ratings of the Helpfulness Potential.

Proposed Intervention rated using a 4 Point Likert scale anchored by 1= probably very helpful and 4= definitely more disruptive than helpful.	MEAN	STANDARD DEVIATION
Establishment of a consistent process or procedure for the "hand off" of diagnostic test results when a provider is absent or leaves the service.	1.58	0.31
A convenient process for providers to generate results letters to patients.	1.59	0.23
A convenient electronic verification when a provider views the diagnostic test result.	1.63	0.21
The establishment of a consistent SOP for results management and reporting by each clinical service.	1.68	0.24
Establishing the expectation for patients that all test results will be reported to them.	1.76	0.07
Periodic summary reports of patients with abnormal test results that have not received the anticipated clinical response (e.g. abnormal mammograms or elevated PSA).	1.78	0.24
A secure voice messaging system to patients for results reporting and instructions from providers.	2.02	0.17
Providing copies of all diagnostic test results directly to patients.	2.03	0.09
Providing, to the ordering service, summary monthly reports of abnormal labs specific to a diagnosis group (e.g. patients with CAD and LDL>110 or CXR with possible mass).	2.07	0.10

### Comments

Almost a third of the VA Primary Care clinicians, practicing in diverse clinical settings, encountered one or more patients with clinically important treatment delays as a result of missed results during the two weeks prior to administration of our survey. Imaging studies and studies related to potential malignancies were the most common types of studies reported missed and cancer was the most common diagnosis which was delayed. Almost 7 per cent of visits to Primary Care were to help patients get results of tests ordered by specialty services with almost half of the providers indicating they often did not know the clinical significance of the results they were asked to research. Significant variation in the processes used to ensure follow up of abnormal results were also reported. Despite practicing in a single healthcare system with a single EMR, providers reported significant variation in the procedures they used to ensure review of ordered diagnostic studies. Finally, respondents reported strong support for a number of potential interventions designed to assist them in managing test results.

These findings add to the growing body of evidence documenting medical errors due to missed diagnostic tests. This study expands upon prior work by providing a more comprehensive picture of both the incidence of missed results in ambulatory practice and the potential clinical ramifications of this problem. The proportion of cancer delays which were prostate, colorectal or lung cancer matches the proportion reported in a review of VHA tort claims from 1998 through 2004 [2]. The higher frequency of cancers diagnoses in both the tort claims review (66 percent) and this study (35 percent) is likely, in part, due to the more severe consequence for a delayed response to a cancer screening study or lost biopsy report. The survey does not capture the clinically significant abnormalities lost to follow up but never discovered by clinicians, and as a result may therefore be a gross underestimate of the true frequency.

While this study adds to the evidence that missed results are ubiquitous and result in harm to patients, finding a simple solution is likely to be challenging. Ensuring a requested test has been completed and integrated into the plan of care involves multiple steps and multiple individuals [10,17]. A recent study found that on average, a full time clinician is currently responsible for reviewing one thousand test results each week [18]. The vast majority of these results will be normal and most of those that are abnormal do not require any specific clinician response [19]. However, given the volume of information that clinicians both generate and review, it is becoming increasingly clear that more robust solutions are needed.

Data from the current study provide evidence that even a well designed computerized in-box system may not prevent busy clinicians from missing results. Such a finding is not surprising given work psychology research that suggests that the vast majority of individuals will ignore alarms as work volume increases or as alarms sensitivity decreases [20,21]. Finally, much could be learned from the aviation industry about redesign of data presentation and decision making to ensure the data volume clinicians must manage each day is more consistent with human limits [6,22,23].

The study is also helpful because it suggests several potential system issues which may contribute to the loss of abnormal tests results. First, variable processes were used by providers to ensure review and follow up of an abnormal result had occurred. Although the computer can automate functions, such as interfaces between the laboratory equipment and the medical record or delivery of data to clinicians, numerous human steps are still required to ensure the information is integrated in the patient's medical care, the patients are notified, and scheduled for follow up when needed [21,22]. Also the relatively frequent numbers of patient diversions suggest that variation in the processes used to manage results is probably occurring in specialty clinics as well as primary care. Also the strong support from the providers for adoption of standard processes for managing and reporting results also suggests that providers recognize the variable processes a contributing system factor. Finally, the lack of consistent patient notification of all test results prevents the activated patient from acting as a failsafe to ensure all results have been reviewed and acted upon.

Furthermore, the waste associated with poor results management is often hidden, therefore difficult to quantify. This includes direct costs for tests never reviewed and the morbidity and mortality of treatment delay associated with missed results. Other less obvious waste, occurring when patients do not receive their test, are the negative impact on Primary Care clinic access and efficiency when patients ask Primary Care to investigate those tests and find results for the patients. Also, when patients are not given their test results, they are less activated, generally experience lower levels of therapeutic adherence, and poorer outcomes [12,24].

### Limits of the study

There are a number of limitations to this study that should be mentioned. Because we are unable to provide a comparison of responders and non-responders, the response rate of 54 percent introduces the possibility of response bias. Even if we assume the unlikely event that all non-responders encountered no patients with missed results or treatment delays during the study period, the numbers of providers reporting errors are still concerning for both missed results (20 percent) and treatment delays (17 percent). In addition, these findings are based entirely upon provider surveys and we lack chart audits to confirm the missed results that were reported. However, our data are consistent with prior studies, making it unlikely that chart audits would have significantly altered our findings [16,25,26].

While it is also possible that over reporting occurred due to recall of events outside the time window, with providers choosing to report because this is the one chance they have to report these type of errors, prior studies have demonstrated provider recall underestimate errors confirmed with chart audits [27,28]. Furthermore we have not captured the clinically significant missed results which were never recognized. Thus it is possible that the rate of error may be either higher or lower than the estimates provided by this survey.

It is important these findings be replicated in settings outside the VA. The frequency of patient diversion from specialist clinics to primary care for test results may be higher in the VA because VA sub-specialty clinics often meet only once or twice a month. Also the VA has a sophisticated EMR with many of the key tools recommended to facilitate more effective management of test results [26,29]. The rate of missed results and associated treatment delay in systems using a less sophisticated EMR or a paper based record may be higher than what was found is this study.

However it is possible that paper based systems by utilizing standardized processes and procedures to ensure all results have been reviewed may have a lower rate of missed results than what we have found in this study. Furthermore systems converting from paper based medical record to an EMR, if systems do not exist for an electronic signature to record physician review of the test result and monitors which identify results that were never viewed, may experience an increase in missed results if process controls which existed in the paper based system to ensure review of abnormal results are not replicated in some fashion in the EMR based system.

In conclusion, true measurement of the burden of missed results within the population is needed, along with a public monitor; however, such tools may be years away. In the interim, the use of provider surveys can reveal useful information for healthcare systems who wish to monitor and improve the management of test results within their system. System interventions to lower the risk of missed results are needed, and data on provider responses to potential interventions are helping guide our selection of interventions to pilot as we work to reduce the burden of diagnostic errors due to the mis-handling of abnormal test results, i.e. missed results.

## Competing interests

The author(s) declare that they have no competing interests.

## Authors' contributions

PC participated in the design of the study and the statistical analysis. TW conceived of the study, and participated in its design and coordination. Both authors developed, and approved the final manuscript.

## Appendix

Survey questions and the response rate (see Table 2).

**Table 2:** Survey questions and response rate

Items	N responding
1. Indicate your healthcare system.	106
2. In the previous two weeks how many clinic sessions have your practiced?	106
3. How many patients do you see in an "average" session?	105
4. Yesterday, or in your previous clinic session, how many patients were directed to you with the intent that you would inform the patient of the results of a diagnostic study that was ordered by a specialty service?	104
5. In the previous 2 weeks, how many patients have you seen with an "abnormal diagnostic result" that was probably missed by the ordering service and not acted upon? (limit to result either >1 month old or of such a critical nature that a 1 month delay would have been inappropriate)	105
6. Please indicate the type of diagnostic results that had been "missed". Check all that apply:	106
7. In the previous 2 weeks, how many patients did you see who may have had a delay in either diagnosis or treatment due to a "missed diagnostic result" that was overlooked by the ordering service?	106
8. What diagnoses or treatments may have been delayed due to a "missed diagnostic"? Select all that apply:	106
How is your practice generally affected when you are asked to provide results to a patient for diagnostics ordered by a different clinical service? Response choices: 5 point Likert scale anchored with 1= strongly agree 5= strongly disagree	
9. The time lost as a result of investigating the test is very burdensome to my practice.	106
10. Generally I do not know the significance of the diagnostic test (that I am being asked to provide) in the other services treatment plans for the patient.	106
11. Do you have a method to monitor if patients received scheduled follow ups for abnormal test results?	106
12. How do you assure that all test results you order are reviewed? Select the answer that best describes your practice:	106
Please indicate how helpful you believe each of these potential interventions would be to VA patients to decrease the risk of needless patient harm due to "missed results": Response options were a 4 point Likert scale anchored by 1= probably very helpful and 4= definitely more disruptive than helpful	
13. Establishing the expectation for patients that all test results will be reported to them.	106
14. Providing copies of all diagnostic test results directly to patients.	106
15. Providing, to the ordering service, summary monthly reports of abnormal labs specific to a diagnosis group (e.g. patients with CAD and LDL>110 or CXR with possible mass).	106
16. Periodic summary reports of patients with abnormal test results that have not received the anticipated clinical response (e.g. abnormal mammograms or elevated PSA).	104
17. Establishment of a consistent process or procedure for the "hand off" of diagnostic test results when a provider is absent or leaves the service.	106
18. The establishment of a consistent SOP for results management and reporting by each clinical service.	102
19. A convenient process for providers to generate results letters to patients.	106
20. A secure voice messaging system to patients for results reporting and instructions from providers.	101
21. A convenient electronic verification when a provider views the diagnostic test result.	103

## Pre-publication history

The pre-publication history for this paper can be accessed here:


